# Magnetic Nanoparticle Mediated Steroid Delivery Mitigates Cisplatin Induced Hearing Loss

**DOI:** 10.3389/fncel.2017.00268

**Published:** 2017-09-13

**Authors:** Bharath Ramaswamy, Soumen Roy, Andrea B. Apolo, Benjamin Shapiro, Didier A. Depireux

**Affiliations:** ^1^Fischell Department of Bioengineering, University of Maryland College Park, MD, United States; ^2^Pfizer Inc. New York, NY, United States; ^3^Sensory Cell Biology, National Institute on Deafness and Other Communication Disorders (NIDCD), National Institutes of Health (NIH) Bethesda, MD, United States; ^4^Genitourinary Malignancies Branch, Center for Cancer Research, National Cancer Institute, National Institutes of Health (NIH) Bethesda, MD, United States; ^5^Institute for Systems Research, University of Maryland College Park, MD, United States; ^6^Otomagnetics Rockville, MD, United States; ^7^Department of Otorhinolaryngology/Head and Neck Surgery, University of Maryland School of Medicine Baltimore, MD, United States

**Keywords:** cisplatin, ototoxicity, magnetic nanoparticles, drug delivery, hair cells

## Abstract

Cisplatin (cis-diamminedichloroplatinum) is widely used as a chemotherapeutic drug for genitourinary, breast, lung and head and neck cancers. Though effective in inducing apoptosis in cancer cells, cisplatin treatment causes severe hearing loss among patients. Steroids have been shown to mitigate cisplatin-induced hearing loss. However, steroids may interfere with the anti-cancer properties of cisplatin if administered systemically, or are rapidly cleared from the middle and inner ear and hence lack effectiveness when administered intra-tympanically. In this work, we deliver prednisolone-loaded nanoparticles magnetically to the cochlea of cisplatin-treated mice. This magnetic delivery method substantially reduced hearing loss in treated animals at high frequency compared to control animals or animals that received intra-tympanic methylprednisolone. The method also protected the outer hair cells from cisplatin-mediated ototoxicity.

## Introduction

Cisplatin (cis-diamminedichloroplatinum) and other platinum based drugs are the antineoplastic drugs of choice for various genitourinary cancers, certain forms of breast cancers, and as radiosensitizers for most head and neck cancers. However, these platinum-based drugs are very toxic to the kidneys (Miller et al., 2010), the inner ear (Rademaker-Lakhai et al., 2006), and sometimes the peripheral nervous system (Gregg et al., 1992). While the nephrotoxicity may be mitigated by hyper-hydrating patients in the hours before and during cisplatin injections, addressing ototoxicity in cisplatin-treated patients remains an unmet medical need.

Cisplatin induced hearing loss occurs in adults with an average incidence of 62% (Marshak et al., 2014). Among pediatric patients, significant sensorineural hearing loss is observed in 90.5% of patients at 8 kHz (Allen et al., 1998). The ototoxic effect of cisplatin is noticeable, with hearing loss within hours or days after the first cisplatin injection (Rybak et al., 2009). It is also cumulative, and the cumulative effect implies particular vulnerability in pediatric populations as repeated treatments, even separated by years, eventually lead to complete hearing loss, which may in turn lead to pervasive developmental delays including speech, cognitive and social developmental challenges.

The molecular mechanisms underlying the ototoxicity of cisplatin remain under debate. The various mechanisms include generation of reactive oxygen species and the depletion of antioxidant enzymes such as superoxide dismutase (DeWoskin and Riviere, 1992), catalase, glutathione peroxidase and glutathione reductase (Rybak et al., 2009). Overall, cisplatin causes damage to the organ of Corti, the stria vascularis and spiral ganglion cells, possibly through different molecular mechanisms (Lee et al., 2004; Monzack et al., 2015).

Steroids have been shown to reduce cisplatin-induced hearing loss, presumably by counteracting the effect of the reactive oxygen species induced by cisplatin administration (Himeno et al., 2002; Marshak et al., 2014). Though commonly used, pre-clinical studies indicate steroids may interfere with cisplatin's efficacy, plus prolonged use of systemic steroids is undesirable due to additive toxicities (Wooldridge et al., 2001; Fardet and Fève, 2014; Morin and Fardet, 2015; Ranganath et al., 2015). Thus, it has been proposed that local administration of steroids into the middle ear, subsequently diffusing into the cochlea via the round window membrane at the base of the cochlea, could be used to protect hearing. However, administration of a liquid steroid into the middle ear results in a rapid elimination of the drug from the cochlea as well as a very steep drug gradient from the base to the apex of the cochlea (Bird et al., 2007; Salt and Plontke, 2009). The liquid formulation in the middle ear is also rapidly eliminated via the Eustachian tube as soon as the patient stands up and swallows.

This paper describes results for protecting hearing from cisplatin by magnetically delivering steroids into the cochlea. In prior animal studies we showed that application of our magnetic injection device could be used to transport drug-eluting bio-compatible nanoparticles from the middle ear to the inner ear. Once inside the inner ear, the drug payload is released from the nanoparticles, providing a significant therapeutic effect (Shapiro et al., 2014a,b). In this paper, we show that magnetic steroid delivery to the inner ear can be used to protect hearing in mice receiving systemic cisplatin regimens. Previously both dexamethasone and prednisolone had been used for their otoprotective effect against cisplatin (Marshak et al., 2014; Özel et al., 2016). We employed prednisolone-loaded magnetic nanoparticles deposited intra-tympanically into the middle ear, and then applied a magnetic field that transported the nanoparticles through the window membranes into the inner ear where they released the steroid in therapeutic amounts. In the mouse model employed in this study, this steroid delivery method effectively mitigated the cisplatin-induced rise in hearing threshold of the animals at high frequencies and protected the outer hair cells in the basal cochlear region from the ototoxic effect of cisplatin.

## Materials and methods

### Animals

The study was conducted on CBA/CAJ mice (10 weeks old) of both sexes (23–27 gm body weight) from the Jackson Laboratory (Bar Harbor, ME). All animal studies were conducted in accordance with the policies and recommendations of the National Institute of Health Guide for the Care and Use of Laboratory Animals, and under approval from the Institutional Animal Care and Use Committee of the University of Maryland.

### Anesthesia

The mice were anesthetized via intraperitoneal injections of ketamine 100 mg/kg and xylazine 20 mg/kg supplemented as necessary and were placed on a warming pad (Deltaphase isothermal pad, Braintree Scientific, MA) to maintain body temperature at 37°C.

### Study design

The overall study design is shown in Table 1. The mouse cisplatin administration protocol (Roy et al., 2013) involves multiple cisplatin cycles spread over time (as is the case for patients), and it reliably elicits hearing loss but leads to less than 10% animal mortality. Compared to this protocol, we halved the duration of the last chemotherapy cycle in an effort to further reduce animal mortality. Hearing of all mice was first tested by auditory brainstem response (ABR) measurements. Then, all mice were pre-hydrated with two subcutaneous doses of 1 mL of sterile normal saline (Hospiral, IL) separated by 8 h, and 24 h before starting each cisplatin cycle, to protect their kidneys against nephrotoxicity. Cisplatin was administered intraperitoneally at 4 mg/kg daily for 4 days on and 10 days off (14-day cycles for the first 2 cycles) plus a 16-day (3rd) cycle (2 days on and 14 days off) (Table 1). During the recovery periods, the animals were hydrated with 1 mL of normal saline twice per day for 5 days or more based on animal's weight and health. After the third cycle, hearing was measured by post-treatment ABR. Then the mice were sacrificed and prepared for cytocochleograms as described below.

**Table 1:** Animal groups and schedule for our cisplatin and ear treatment study.

	**Group A: Saline control**	**Group B: Intra-tympanic methyl-prednisolone**	**Group C:Magnetic delivery of nanoparticles without prednisolone**	**Group D: Magnetic delivery of nanoparticles with prednisolone**
**Number of animals**	***N* = 6**	***N* = 6**	***N* = 4**	***N* = 6**
Day 0	Pre-treatment auditory brainstem response recording			
Day 1–4	4 mg/kg of cisplatin per day	4 mg/kg of cisplatin per day	4 mg/kg of cisplatin per day	4 mg/kg of cisplatin per day
Day 5–14	Recovery period with 2 mL/day of subcutaneous saline injection			
Day 15	Left ear saline injection	Left ear methyl-prednisolone injection	Left ear nanoparticle injection + magnet exposure for 20 min	Left ear nanoparticle injection + magnet exposure for 20 min
Day 16–19	4 mg/kg of cisplatin per day	4 mg/kg of cisplatin per day	4 mg/kg of cisplatin per day	4 mg/kg of cisplatin per day
Day 20–29	Recovery period with 2 mL/day of subcutaneous saline injection			
Day 30	Left ear saline injection	Left ear methyl-prednisolone injection	Left ear nanoparticle injection + magnet exposure for 20 min	Left ear nanoparticle injection + magnet exposure for 20 min
Day 31–32	4 mg/kg of cisplatin per day	4 mg/kg of cisplatin per day	4 mg/kg of cisplatin per day	4 mg/kg of cisplatin per day
Day 33–46	Recovery period with 2 mL/day of subcutaneous saline injection			
Day 47	Post-treatment auditory brainstem response recording			
Day 50	Termination and cochlear preparations			

The animals were divided into four different groups, with *N* = 6 mice per group for group A, B and D and *N* = 4 mice for group C. For all groups, ear treatment was administered 1 day before the second and third cisplatin cycles respectively. Group A mice received 1.8 μL of intra-tympanic saline into their left ears. Group B mice received 1.8 μL of intra-tympanic methylprednisolone (Pharmacia&Upjohn, NJ) at a concentration of 40 mg/ml (close to the clinical dosage used in humans) into their left ears. Group C mice received 1.8 μL of 300 nm diameter magnetic nanoparticles without prednisolone into their left ears. Group D mice received 1.8 μL of 300 nm diameter magnetic nanoparticles that were loaded with prednisolone sodium phosphate at a concentration of 82 μg/mL (the maximum drug loading these particles could carry), also into their left ears. Both unloaded and prednisolone loaded particles were labeled with Texas red fluorescent dye for easy visualization in tissue samples (Chemicell, Berlin, Germany). A 0.5 Tesla magnet (5 × 2.5 × 2.5 cm, K&J Magnetics, PA) was then placed contralateral near the right eye of each animal in group C and group D for 20 min to pull the nanoparticles from the middle ear into the inner ear. For all animals in all groups, the right ears remained as untreated same-animal controls.

### Auditory brainstem response

**Figure 1:**
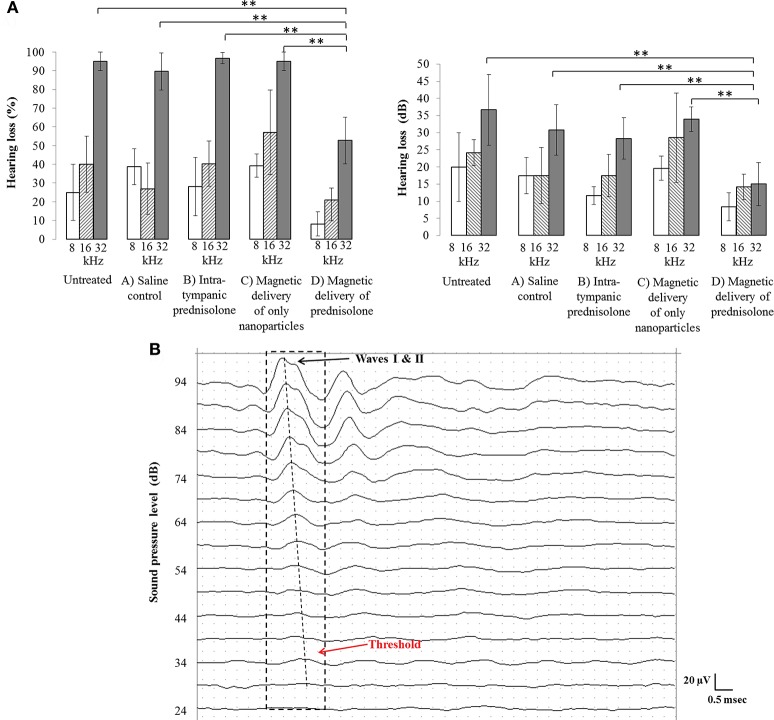
**(A)** Comparison of hearing loss between the four animal groups for the injected left ears and corresponding untreated right ears. Percent hearing loss at each frequency (Left) and the corresponding hearing loss in dB (Right), per group, are shown. Treated ears that received magnetic nanoparticles showed significantly less hearing loss (group D bars), as compared to the only nanoparticle group (group C bars, ^**^p < 0.05), the intra-tympanic methyl-prednisolone group (group B bars, ^**^p < 0.05), and the saline control group (group A bars, ^**^p < 0.05), especially at the high frequency of 32 KHz. Hearing loss remained similar at high frequency across all groups for untreated ears. **(B)** A sample ABR trace containing the waves I and II (dotted black box) at 16 KHz has been shown, to demonstrate the threshold measurement (note that positive voltage is up, the convention for animal ABRs). The threshold for this animal is at 34 dB beyond which the waves I and II are completely attenuated.

The hearing thresholds of the animals in all groups were measured by performing auditory brain stem response (ABR) assays before and after the cisplatin treatment and recovery periods. The mice were anesthetized and placed inside a sound booth (Industrial Acoustics, NY). Two recording electrodes (RLSND110-1.5, Rhythmlink International) were inserted postero-ventral to the auricular area of the left and right ears. A reference electrode was placed at the apex of the head. A ground reference electrode was placed subcutaneously in the lumbar area. Using our ABR recording system (Tucker Davis Technologies, FL), the animals were then presented in free field with 600 sweeps of 5 ms long bursts (shaped with 1 ms onset and offset sinusoidal ramps) at varying intensities beginning at a 94 dB sound pressure level (SPL) and proceeding in 5 dB decrements down to a 14 dB SPL. The electrophysiological signals were recorded for 10 ms. These cycles of sound intensities were repeated for different sound frequencies (8, 16, and 32 kHz). Hearing threshold at each frequency was determined as the lowest intensity at which a definite cochlear response could be identified (waves I & II). Figure 1B shows sample traces at 16 KHz for various SPLs and the corresponding hearing threshold. The percentage hearing loss of each animal at a specific frequency was defined as the ratio of the change in thresholds after the treatment compared to pre-treatment thresholds. Hence 0% represents no loss in hearing at that frequency (pre and post hearing thresholds were identical), while 100% represents no measurable response or a measurable response only at the highest sound pressure level of 94 dB.

### Cytocochleogram

The cochleas from the different groups were dissected to study the pathophysiology of the cisplatin treatment on the organ of Corti, as well as any effect of the otoprotective treatments. The animals were euthanized using carbon dioxide and the cochleas rapidly isolated. The cochleas were continuously perfused with ice cold 4% paraformaldehyde into the round window membrane and out of a small hole pierced in the apex of the cochlea, and then placed in paraformaldehyde overnight at 4°C. This was followed by 3X wash with 1X PBS at pH 7.4 and decalcification in 0.5 M EDTA for 3–4 days. After washing the cochleas three times using 1X PBS, they were micro-dissected into three turns (Basal, Middle, Apical) using an ophthalmic knife (MANI Ophthalmics, Tochigi, Japan). The tectorial membrane was removed. Cochlear outer and inner hair cell layers were stained using Alexa Fluor 488 Phalloidin (1:800 in 1X PBS + 0.5% Tween, Life Technologies) for 45 min. The turns were mounted on a glass slide using Fluoromount-G with DAPI (Electron Microscopy Sciences). Images of each cochlear turn were taken at 40X magnification using an LSM 710 confocal microscope (Zeiss) in z-stack mode. The outer hair cells in these images were counted for the presence of nuclei and cell membranes over a 200 μm distance of the different cochlear turns using Zen 2010 software (Zeiss) and the assessments were made blind to avoid biases.

## Results

In our experiments, the hearing thresholds of the animals in the different groups were determined after the completion of the cisplatin treatment by using ABR assays. The hearing loss experienced by the animal at a particular frequency was determined by the ratio of change in threshold post-treatment to the initial hearing threshold at the same frequency. After systemic cisplatin treatment, this threshold increase is known to occur first at high frequencies, progressing to the lower frequencies as treatment continues, eventually reaching speech frequencies (Rybak et al., 2009; Chirtes and Albu, 2014).

In our study, for untreated (right) ears the hearing loss was greatest at high frequency (at 32 kHz), as compared to at 16 and 8 kHz (Figure 1A). In treated (left) ears at 32 kHz, the magnetic delivery group D ears experienced substantially less hearing loss (53 ± 12%) compared to ears that received saline (group A, 93 ± 7%) or to intra-tympanic methylprednisolone (group B, 97 ± 3%) or to only magnetic nanoparticles (group C, 95 ± 5%). The error bars indicate the standard deviation observed in the measurements. As evident in Figure 1A at 32 kHz, the difference in means between group D and the other groups was much larger than the variance within each group. According to a standard *t*-test, magnetic delivery achieved a statistically significant reduction in high frequency hearing loss for the magnetically treated group D (magnetic delivery of prednisolone) ears, as compared to control group A, B, and C ears which exhibited almost complete hearing loss at high frequencies (^**^p < 0.05). Overall, the prednisolone loaded nanoparticles mitigated cisplatin induced ototoxicity at high frequencies, with 95% statistical significance. At 8 and 16 kHz, magnetic delivery also seemed to reduce the degree of hearing loss, but at these lower frequencies a statistical significance of 95% was not reached, in part because cisplatin caused less hearing loss in mice at these lower frequencies (see the 8 and 16 kHz bars in Figure 1A). A greater effect at high frequencies may also be because the drug coated nanoparticles have easier access to the basal layer of the cochlea, which corresponds to high frequencies of hearing.

**Figure 2:**
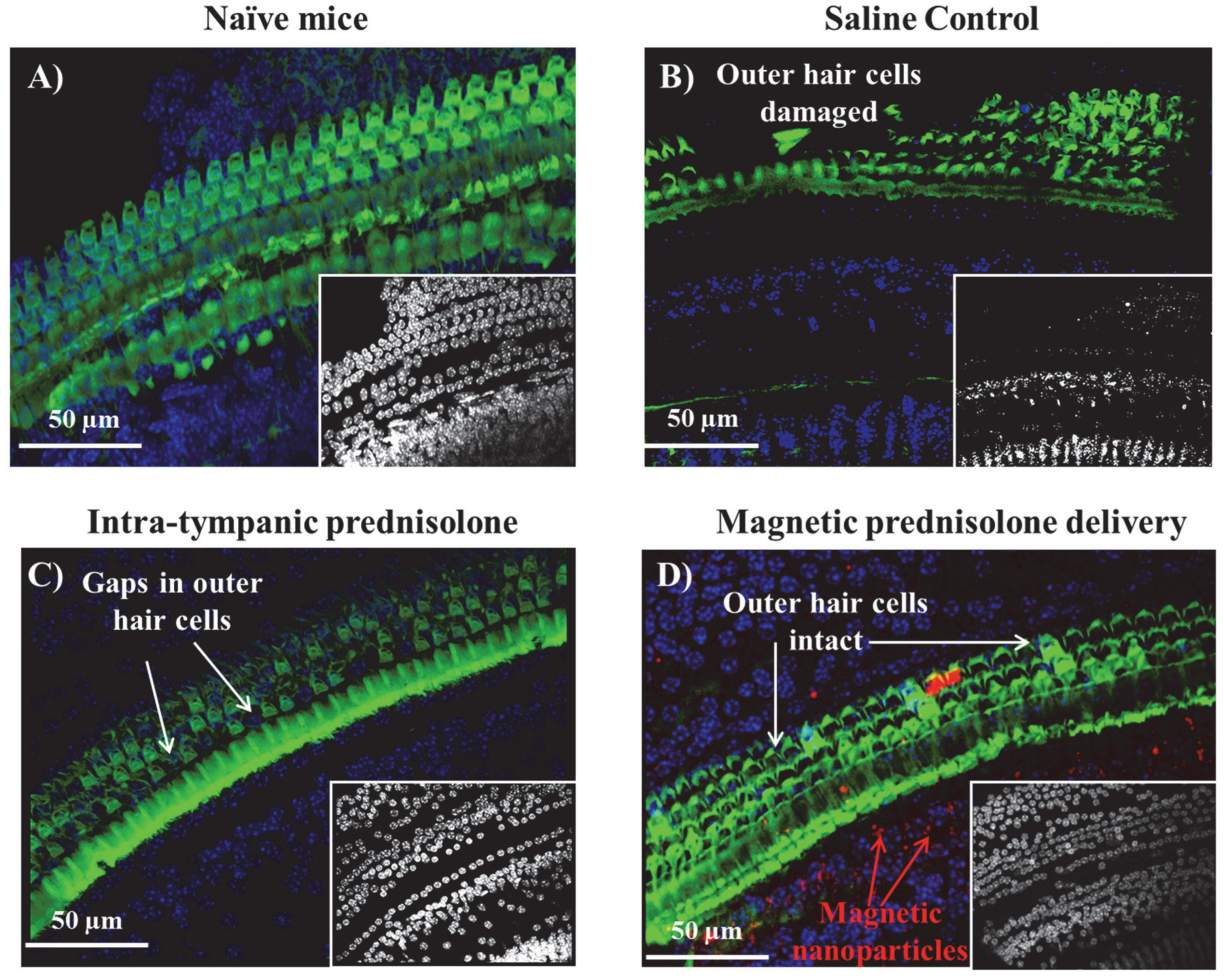
Sample cytocochleograms of the basal cochlear region of different groups. The outer hair cells were stained for actin with Alexa Fluor 488 Phalloidin (green) and the various cell nuclei were stained using DAPI counterstain (blue). **(A)** Left ear from a naïve animal that did not receive any cisplatin treatment or otoprotection. For animals that were administered cisplatin: **(B)** Left ear that received saline; **(C)** Left ear that received intra-tympanic methyl-prednisolone; and **(D)** Left ear that received magnetic delivery of prednisolone. The images of the DAPI stained nuclei for all the groups have been shown in the image insets of **(A–D)** correspondingly. (A version of this figure has also appeared in an invited feature article in The Hearing Journal, July 2017 issue.)

Cisplatin is known to induce apoptosis in the three rows of outer hair cells starting at the outer row and progressing to the inner row (Kujawa and Liberman, 2006). There have also been reports of damage to the inner hair cells of the organ of Corti, cuticular plate and stria vascularis (Chirtes and Albu, 2014). These ototoxic effects have been consistently shown to progress from the basal, high frequency region of the cochlea to the apical, low frequency region with continuing cisplatin treatment. We extracted cytocochleograms to evaluate the effect of magnetic delivery in protecting hair cells. The cochleas were micro-dissected post-treatment and the organ of Corti examined in the different turns after staining the hair cells. Sample cytocochleograms are shown for the basal cochlear region (from the window membrane to the distal end of the cochlea) in Figure 2. Hair cell preservation is evident in the magnetically treated cochlea (Figure 2D) compared to groups that received intra-tympanic saline or methyl prednisolone injections. Magnetically delivered nanoparticles can be seen among the hair cells in the cochlea (red fluorescence in Figure 2D).

The number of outer hair cells in each cochlear micro-sections was counted and compared between the cochleas receiving magnetic treatment, receiving intra-tympanic saline, and receiving methyl-prednisolone injections. The outer hair cell density observed for control animals (no cisplatin and no ear treatments) vs. from ears of animals that received cisplatin plus one of the three types of ear treatments (saline, intra-tympanic methylprednisolone, or magnetic delivery of prednisolone) is shown in Table 2. A significant decrease in hair cells was observed for the saline ears (72% decrease) and intra-tympanic methyl prednisolone ears (33% decrease). In contrast, cochleas from treated ears in the magnetic delivery group displayed a small loss of 9% of hair cells in the basal region compared to control (no cisplatin) animals. This indicates that in the magnetic delivery of prednisolone group, the outer hair cells in the basal cochlear region were preserved.

**Table 2:** Comparison of outer hair cell density for cochleas in naïve mice (*N* = 6), vs. in mice that received the three ear treatment types (also *N* = 6 for each group). The second row lists the percent decrease in hair cell density compared to the no cisplatin naïve group. In the magnetically treated group D, hair cell density decreased by just 9% compared to substantially greater hair loss in the other groups.

	**Naïve mice: no cisplatin or ear treatment**	**Treated ears : cisplatin + intra-tympanic saline**	**Treated ears: cisplatin + intra-tympanic methyl prednisolone**	**Treated ears: cisplatin + magnetic delivery of prednisolone**	**Untreated ears: cisplatin, but no ear treatment**
Outer hair cell density (number per 200 μm)	75 ± 2	21 ± 5	50 ± 7	68 ± 4	26 ± 9
% decrease in hair cell density	N/A	72%	33%	9%	65%

**.

## Discussion

The benefit of magnetic delivery is that it actively and directly transports drug into the cochlea, in contrast to intra-tympanic administration where drug diffuses passively into the cochlea from the middle ear. Although it was not possible to measure the amount of steroid delivered into mouse cochleas in this study (the mouse perilymph sample volume was too small to enable mass spectrometry measurement of prednisolone concentrations), in initial adult human cadaver studies we have observed significantly greater delivery of steroid to the cochlea with magnetic delivery as compared to intra-tympanic administration without magnetic fields and particles. Improved delivery to the cochlea, and also to the vestibular system, is of interest not only for protection of hearing from chemotherapy, but also for other conditions of the inner ear such as sudden and noise-induced hearing loss, for presbycusis, for tinnitus, and for Ménière's disease. This study achieved efficacy by delivering prednisolone, a generic off-the-shelf steroid. However, magnetic nanoparticles can be loaded with many other therapies. Magnetic delivery of new and emerging therapies (novel compounds, growth factors, gene therapy) is anticipated to yield even greater benefits than delivery of a generic drug. Conversely, even the best new drug or therapy will not be effective if it does not reach the cochlea in sufficient quantities to be therapeutic.

The cisplatin regimens that were administered to the animals caused hearing loss primarily at high frequencies. In human patients also, hearing loss due to cisplatin is known to occur first at higher frequencies and then to progress to lower frequencies as treatment continues (Rybak et al., 2009; Chirtes and Albu, 2014). Magnetic delivery of prednisolone acted primarily to protect this high frequency hearing, as evident in the right panel of Figure 1A where the 32 kHz hearing loss for untreated ears and group A-C ears was reduced in group D ears. Greater protection at high frequency may be due both to the fact that there was more hearing to be saved at higher than at lower frequencies, and perhaps also because particles are delivered through the window membranes first to the base of the cochlea (which is where high frequency hearing resides). Intra-tympanic prednisolone treatment without magnetic particles did decrease cochlear outer hair cell loss (Figure 2C), but high frequency hearing was still lost in this group (Figure 1, group B). Such a mismatch between outer hair cell loss and hearing loss has been observed previously in the literature (Kujawa and Liberman, 2006) and may be due to other factors such as damage to spiral ganglion neurons or stria vascularis cells.

## Conclusions

Cisplatin administration is known to be ototoxic, likely by the production of reactive oxygen species in the inner ear and by depletion of the inherent antioxidant system of the cochlea, leading to apoptosis of hair cells in the organ of Corti, spiral ganglion cells, and marginal cells of the stria vascularis (Boulikas and Vougiouka, 2003; Chirtes and Albu, 2014). Steroids such as dexamethasone and prednisolone are thought to reduce the production of free radicals in the inner ear and decrease the formation of inflammatory molecules and could protect hearing from cisplatin (Marshak et al., 2014; Okano, 2014; Özel et al., 2016). However, prolonged systemic administration of steroids may reduce the anti-cancer efficacy of cisplatin and is also undesirable due to added toxicity (Wooldridge et al., 2001; Herr et al., 2003; Zhang et al., 2006; Fardet and Fève, 2014; Morin and Fardet, 2015; Ranganath et al., 2015), which has led to studies on local intra-tympanic administration of steroids to protect hearing (Herr et al., 2003). Compared to intra-tympanic administration, magnetic forces can better deliver therapy directly to the cochlea and can confer a stronger therapeutic effect. In this animal study we observed that magnetic delivery of steroid protected hair cells more effectively and concomitantly reduced the degree of cisplatin-induced hearing loss; as compared to no treatment, to intra-tympanic steroid administration, and to magnetic delivery of nanoparticles without attached steroid.

## Author contributions

All authors contributed to the research presented in this article. BR conducted the experiments. SR provided guidance on the cisplatin ototoxicity animal model. AA provided guidance for clinical needs. BS and DD provided guidance on design of experiments and the magnetic delivery system. All authors contributed to writing the article.

### Conflict of interest statement

DD and BS disclose that they have an ownership interest in Otomagnetics (including patents). The other authors do not have any financial relationship to the research to disclose. The other authors declare that the research was conducted in the absence of any commercial or financial relationships that could be construed as a potential conflict of interest.
